# Diagnostic and Therapeutic Approach in a Patient with Buerger's and Coronary Artery Disease

**DOI:** 10.1155/2013/974184

**Published:** 2013-11-28

**Authors:** Fotios Mitropoulos, Fotios Eforakopoulos, Meletios A. Kanakis, Maria Vassili, Irene Mastorakou, Michael Georgiadis

**Affiliations:** Department of Pediatric and Congenital Heart Surgery, Onassis Cardiac Surgery Center, 356 Syngrou Avenue, 176 74 Kallithea, Greece

## Abstract

Occlusive coronary artery disease coexisting with Buerger's disease has rarely been reported. Potential difficulties regarding diagnostic workup and therapeutic management in this group of patients are discussed through this case report. We present an interesting case of a 52-year-old patient suffering from Buerger's disease, with a history of generalized peripheral occlusive arteriopathy, who presented with acute coronary syndrome. A difficulty in accessing and performing coronary angiography was evident due to the vascular status of the patient. Diagnosis was performed by computed tomography (CT) of the coronary arteries. It showed 80–90% obstruction of the LAD, and since percutaneous coronary intervention was impossible, a single aortocoronary bypass grafting was performed with the off-pump technique. Coronary artery disease coexisting with Burger's disease is a rare entity, and CT angiography is a useful diagnostic tool, when the classic angiography could not be performed. In addition, off-pump coronary artery bypass should be the therapeutic option of choice in this high risk group of patients. The uncomplicated postoperative course of the patient and his hitherto good condition showed that both diagnostic and therapeutic procedures were the best possible.

## 1. Introduction

Thromboangiitis obliterans (TAO or Buerger's disease) is one of the major causes of peripheral vascular disease and is related to smoking. It is a nonatherosclerotic, segmental, and inflammatory vasculitis, during which cellular clots are being generated and cause obstruction of the blood vessels, while relatively respecting the structure of the mural surfaces [1]. Although it frequently affects the vessels of upper and lower extremities, coronary artery involvement is very unusual and rarely reported [2–4].

An unusual case of a patient with Buerger's disease with coronary artery disease is described, and special emphasis on the diagnostic and therapeutic issues is discussed.

## 2. Case Presentation

A 52-year-old patient, heavy smoker with hypertension and COPD, has been suffering from thromboangiitis obliterans (TAO) for the last 25 years. Within the frame of symptomatic treatment of TAO, lumbar sympathectomy was followed by spinal cord stimulator.

His amputation history is impressive as it includes right lower limb amputation, at the level below knee; epicondylar amputation of the left extremity; amputations of the left index, of the right middle finger, and of the right little finger. His vascular history includes 60% stenosis of the right internal carotid artery, abdominal aortic aneurysm (43 mm), 40% stenosis of the left renal artery, and a significant degree of stenosis of the iliac vessels (40% on the left and 60% on the right). Pulses were absent from the femoral, the radial, and ulnar arteries, on both sides. The patient had never stopped smoking.

He was diagnosed with an anterior ST segment elevation myocardial infarction (STEMI). Cardiac echocardiography revealed left ventricular dilatation with severely impaired contractility overall with myocardial akinesis of the interventricular septum of apical and inferior segments. Ejection fraction was 25%. He was then referred for coronary angiography.

**Figure 1:**
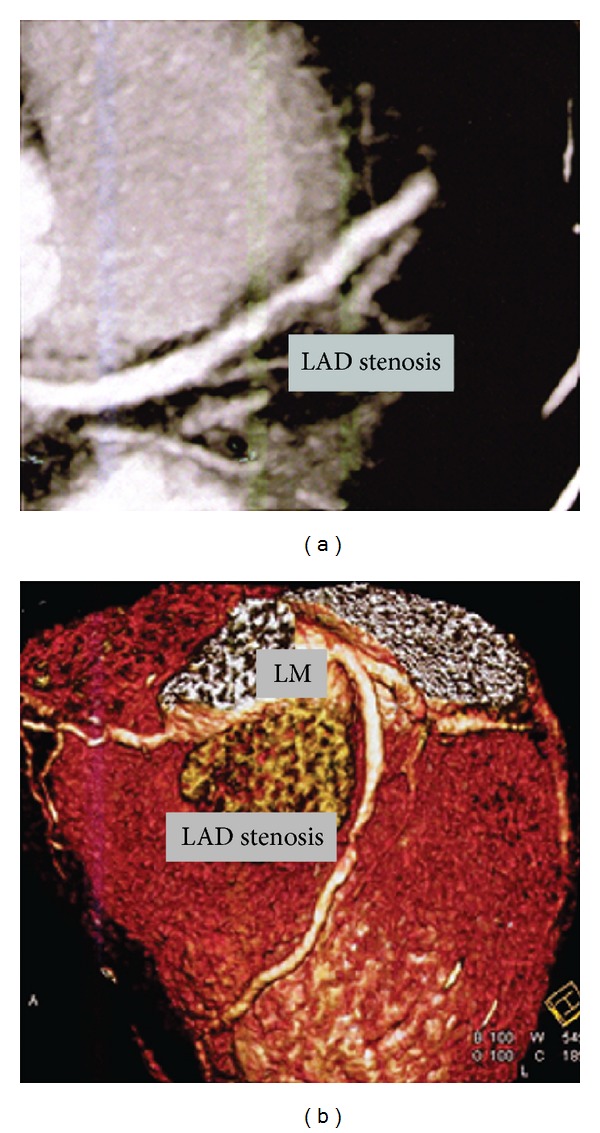
Atherosclerotic lesion of proximal part of the left anterior descending (LAD: left anterior descending and LM: left main coronary artery).

A difficulty in accessing and performing coronary angiography was evident due to the vascular status of the patient. Since he had no clinically peripheral pulses, the decision was made to proceed with computed tomography (CT) of the coronary arteries. It showed 80–90% obstruction of the LAD (Figures 1(a) and 1(b)). He was then referred for surgical treatment.

A single aortocoronary bypass grafting was decided and performed. During the operation, pedicled graft of left internal mammary artery (LIMA) was anastomosed in situ distally to the injury of the LAD, without using extracorporeal circulation (Off-pump), given the high risk of complications that this would entail. The flow from LIMA was very satisfactory. He was transferred to ICU with moderate inotropic support. He was extubated on the first postoperative day and transferred to the ward on the third postoperative day without inotropic support. On the 8th postoperative day, he was discharged in a generally good condition. There were no neurological, renal, or respiratory complications. 12 months after the operation is symptom-free, he has stopped smoking and ejection fraction is of 45%.

## 3. Discussion

Buerger's disease (TAO) is a nonatherosclerotic, inflammatory disease affecting the small and medium-sized arteries and veins of the extremities. It is mainly found in the Mediterranean area, as well as the Middle East and Far East. In Europe, 4%-5% of hospitalized people are affected by peripheral vasculopathy. It typically affects young male patients with onset before the age of 40, resulting in ischemic limbs, and there is a strong correlation with smoking [5–7].

There are no specific laboratory tests to aid in the diagnosis of Buerger's disease. The pathologic findings depend on the duration of the disease [7]. Early lesions are characterized by intravascular clots and infiltration of neutrophils, with a small involvement of the vessel wall, and they are pathognomonic. In chronic conditions, a gradual organization of the thrombus is developed, with infiltration of monocytes and epithelioid cells, while, at the same time, fibrosis is formed. Regardless of the stage of disease, the vessel wall remains intact, which in fact separates TAO from atherosclerosis and other forms of vasculitis [7].

Clinical presentation includes intermittent claudication, phlebitis migrans, rest pain, ischemic ulcers of the limbs, and eventually gangrenes. Diagnostic workup includes angiogram which can exclude any other reason of limb ischemia, while it may demonstrate the typical findings of TAO: obstruction of the small and medium-sized vessels, with the development of collateral spiral vessels without the involvement of the proximal portions of the limbs [5–7].

Although Buerger's disease generally involves the distal extremities, it has been reported in other vessels, including the coronary arteries. Mautner and colleagues reviewed 13 published cases of Buerger's disease in which the coronary arteries were studied histologically. Atherosclerosis was the predominant histological finding, with the coexistence of lesions consistent with Buerger's disease [8].

In our case, severe peripheral obstructive vascular disease did not allow the conventional angiographic workup. To the best of our knowledge, it is the first time in the literature research that the involvement of coronary vessels during Buerger's disease is depicted with the use of coronary angiography.

CT angiography is a relatively new imaging method where by the use of multivolume CT scanner and through the administration of intravenous contrast, rapid scanning of the myocardium is performed, always in direct correlation with the cardiac cycle. Using that method, we manage to test the lining of blood vessels, atherosclerotic lesions, the degree of calcification of the plaque (soft, calcificated, and plaque with a mixed texture), various congenital anomalies, and the anatomy of the valves and other structures of the thoracic cage. The sensitivity of the method ranges from 95% to 99% and its specificity from 95% to 98%, and it represents a reliable method for excluding or proving coronary artery disease [9].

In our case, the test was carried out by the use of a machine SOMATOM Definition, consisting of two light lamps and 64 intersections. Image processing was followed by MPR (multiplanar reconstruction) techniques, MIP (max intensity projection), and VRT (volume rendering technique). After scanning that was done under gated ECG guidance, beam focusing, collimation of 0.6 mm, and reconstruction of 3 mm, a low load of Ca^+2^ was found. The proximal part of LAD, a plaque consisting of a mostly soft texture, rises, causing blockage by 80–90%, while the rest of the vessels presented minimum stenoses. This fact is consistent with coronaries' involvement by Buerger's disease, in which the vessels' wall is not seriously damaged. The patient's history, the involvement of internal carotid and visceral vessels, mild coronary atherosclerosis, and the solitary lesion of soft structure with a low degree of calcification, allows us to say with a relative safety that this case could be a rare case of coronaries' involvement by TAO. However, the definite diagnosis would be given only by histological examination of the artery.

Considering the status of the patient in conjunction with low ejection fraction, the decision was to carry out coronary artery bypass grafting, without the use of extracorporeal circulation (OPCAB) since for access reasons, the PCI was excluded.

In conclusion, coronary artery disease coexisting with Burger's disease is a rare entity and CT angiography is a useful diagnostic tool, when the classic angiography could not be performed. In addition, OPCAB should be considered as the appropriate therapeutic option in this high risk group of patients.
